# Potential of Novel EPO Derivatives in Limb Ischemia

**DOI:** 10.1155/2012/213785

**Published:** 2012-02-22

**Authors:** Dhiraj Joshi, Janice Tsui, Rebekah Yu, Xu Shiwen, Sadasivam Selvakumar, David J. Abraham, Daryll M. Baker

**Affiliations:** ^1^Vascular Unit, Division of Surgery and Interventional Science, Royal Free Hospital, University College London (Royal Free Campus), Pond Street, London NW3 2QG, UK; ^2^Centre for Rheumatology, University College London (Royal Free Campus), Pond Street, London NW3 2QG, UK

## Abstract

Erythropoietin (EPO) has tissue-protective properties, but it increases the risk of thromboembolism by raising the haemoglobin concentration. New generation of EPO derivatives is tissue protective without the haematopoietic side effects. Preclinical studies have demonstrated their effectiveness and safety. This paper summarizes the development in EPO derivatives with emphasis on their potential use in critical limb ischaemia.

## 1. Introduction

Critical limb ischaemia (CLI) causes considerable morbidity and mortality. Surgical and endovascular revascularization are the main treatment options. One-fourth of patients presenting with CLI undergo a major amputation, primarily because they are not suitable for revascularization [1]. Pharmacotherapy has negligible role in the management of CLI. Prostacyclin is the only disease-modifying drug with proven efficacy in reducing rest pain and/or ulcer size in CLI via its vasodilatory effects on the distal circulation [2]. The Trans-Atlantic Inter Society Consensus document on peripheral vascular disease (TASC) has recommended the use of iloprost in CLI to avoid or delay major amputation [3]. There is limited evidence supporting the use of other vasoactive drugs such as pentoxifylline, cilostozol, and L-Arginine (precursor of nitric oxide) in the treatment of CLI [4].

More recently, the possibility of using recombinant formulations of growth factors to augment the development of collaterals and new capillaries from pre-existing blood vessels has been explored. This is otherwise known as therapeutic angiogenesis [5]. Prominent among these techniques is the intra-arterial or intramuscular administration of vascular endothelial growth factor (VEGF) plasmid DNA (phVEGF_165_) [6] where initial studies in patients with CLI demonstrated an increase in angiogenesis [7]. Other than gene therapy, clinical studies have been carried out to inject bone-marrow mononuclear cells intramuscularly in CLI. The innate ability of bone marrow cells to supply endothelial progenitor cells and release endothelial growth factors and cytokines also helped induce angiogenesis [8]. However, despite a demonstration of increased angiogenesis, improvement in clinical endpoints was less clear. This is likely to be due to the complex pathology involved within the ischaemic tissue [9].

Tissue protection may be an alternative therapeutic strategy for CLI where reducing tissue damage and maintaining tissue viability may improve outcome of current treatments and contribute to limb salvage. It has been shown previously that limb ischaemia causes apoptosis of skeletal muscle [19]. Therefore, the use of tissue protective agents to decrease apoptosis and inflammation, either alone or in combination with other therapeutic strategies, may be beneficial.

Erythropoietin (EPO) has tissue protective properties that have been described extensively [20, 21]. We have recently shown that there is expression and upregulation of EPO receptors (EPORs) in human skeletal muscle [18]. However, EPO triggers profound haematopoiesis that increases the risk of thromboembolism [22]. This may prevent its use in CLI. Lately, nonhematopoietic EPO derivatives have been developed, which avoid its hematopoietic side effects [23]. The aim of this paper is to summarize the developments that have taken place so far in the use of EPO derivatives in protection of ischaemic tissue and discuss its potential use in CLI.

## 2. EPO and Its Receptors

**Figure 1:**
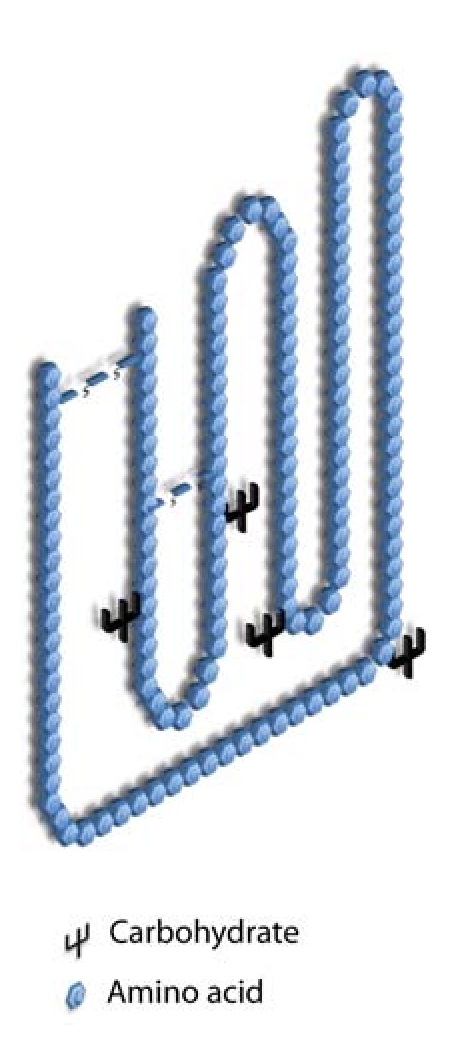
A schematic representation of the molecule of EPO. Adapted from Elliott et al. [25]

Erythropoietin (EPO) belongs to the class-I cytokine family. It is a glycoprotein hormone with a molecular mass of 30.4 kDa [24]. The protein core contains 165 amino acids and the peripheral carbohydrate chains are made up of 4 N-linked oligosaccharides (Figure 1) [25].

**Figure 2:**
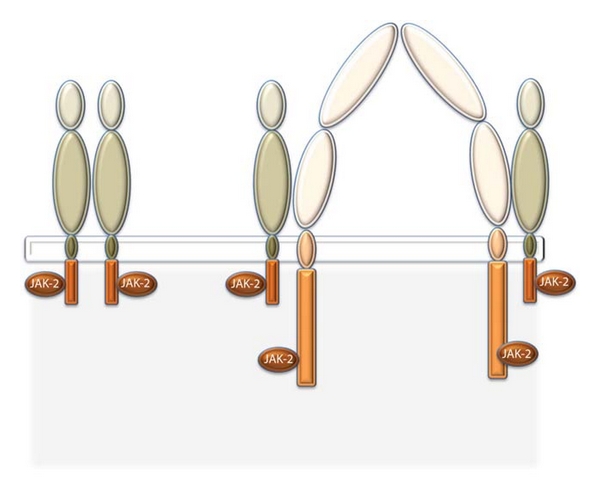
Schematic representation of the structure of erythropoietin receptor (EPOR) and beta-common receptor (CD131) on the left and the right hand side, respectively. *Based on Brines et al. [29]. JAK-2 stands for Janus family Tyrosine Protein Kinase 2.

Human EPO receptor (EPOR) is a cell surface protein containing 508 amino acids and has a molecular weight of 55 KDa [26]. It belongs to the cytokine receptor superfamily and contains extracellular, transmembraneous, and intracellular domains [27]. The common *β*-chain of IL-3/IL-5/GM-CSF receptor (also called CD131 or *β*-common receptor) belongs to the same super-family. The molecular weight of *β*-common receptor (*β*cR) is approximately 130 kDa [23]. The EPO molecule binds to two EPOR subunits (EPOR dimers or (EPOR)_2_) to bring about the haemopoietic action. In contrast, the EPO molecule binding to two *β*cR subunits sandwiching two EPOR subunits mediates the tissue protective function [23]. The four subunits constitute the heteroreceptor complex, which binds to the EPO molecule and activates cell signalling (Figure 2). The intracellular or cytoplasmic domain of all cytokine receptors is intangibly related to a Janus family tyrosine protein kinase 2 (JAK2). The binding of EPO to its receptor (EPOR homodimer or *β*cR and EPOR) induces a conformational change in the receptor, activating a downstream signalling pathway that leads to the transcription of new proteins that determine the fate of the cell [28].

Endogenous EPO is essential for the maturation and survival of erythrocytes and thus maintaining the normal haemoglobin concentration. In chronic renal failure, the kidneys cease to make EPO, and commercially available preparations of recombinant human EPO (rHuEPO) are mainly used to treat anaemia secondary to chronic renal failure [30]. In addition to its role in prolonging the survival of erythrocytes, EPO has been shown to exert anti-apoptotic and anti-inflammatory effects in neural and cardiac tissue both *in vitro *and* in vivo*. Clinical trials have also confirmed its beneficial effects in stroke and myocardial infarction [21, 31].

However, the ability of rHuEPO to bond with EPOR in extrahaematopoietic tissues including vascular endothelium is about 50 times less than that in the erythroid cells [32]. Hence, the dose of EPO required to attain a tissue protective effect in heart and brain ischaemia is much higher than that used for the management of anaemia in renal failure. In CLI, the volume of ischaemic tissue is even greater than that in heart and brain ischaemia, therefore higher doses still may be required to achieve a therapeutic effect. This will cause an unfavorable increase in the haematocrit and blood viscosity, which in turn will raise the risk of thromboembolism and occlusion of arteries with pre-existing disease [33, 34]. Another concern is a potential effect on tumour progression due to its angiogenic properties [35]. However, recent evidence suggests that this may not be a significant risk [36]. EPO also causes hypertension by its action on vascular smooth muscle cells [37–39].

Due to the potential side effects, most research on the tissue protective effects of EPO in CLI and other tissues has been limited to preclinical models. However, the latest nonhaemopoietic EPO derivatives may overcome the unnecessary side effects and permit the translation of preclinical results into clinical trials [11]. 

## 3. Nonhaematopoietic Derivatives of EPO

### 3.1. Carbamylated Erythropoietin (CEPO)

 CEPO is formed by the chemical modification of the amino acid lysine to homocitrulline. It changes the receptor-binding capability of the EPO molecule [11].

### 3.2. Asialo EPO

Asialo EPO is formed by decreasing the sialic acid content of rHuEPO by a technique known as the total enzymatic desialylation. Remarkably, the molecule retains its tissue protective property but loses the haemopoietic ability. This also results in the reduction of the half-life of the Asialo EPO to minutes [10].

### 3.3. Helix-B Surface Peptide (HBSP) [40]

 The EPO molecule, a glycoprotein that contains 165 amino acids is folded in such a way that the hydrophobic areas-helixes A, C, and D face away from the surface and are embedded in the extracellular domains of (EPOR)_2_. However, helix B that is hydrophilic points away from the receptor and has wide-ranging tissue-protective properties. Isolating the helix B which is devoid of the primary sequence resulted in a low molecular weight peptide. The spontaneous cyclization of Helix B surface peptide (HBSP) gives rise to pyroglutamate HBSP (pHBSP), which is also known as ARA 290. The effective dose of ARA 290 peptide that showed evidence of tissue protection was comparable on a molar basis to those studied for EPO and was higher than that required for EPO-mediated erythropoiesis. For example, in the renal ischaemia model of mice, 0.08 nmol/kg of bw (equivalent to 300 units/kg of body weight of EPO) was ineffective, whereas a 10-fold higher dose elicited strong tissue protection [40]. The studies of non-erythropoietic EPO derivatives have been summarised in Table 1.

## 4. Mechanism of Action

Receptor-initiated cell signaling by EPO and its derivatives occurs via multiple molecular cascades, which differ in importance depending on the specific tissue or cell type, as well as the type of injury [41]. As in erythropoiesis, the majority of tissue-protective responses begin by the phosphorylation of JAK-2 or JAK-1 [42, 43]. Following JAK-2 phosphorylation, 3 main pathways are activated that result in attenuation of apoptosis: STAT-5/Bcl-xL (Signal Transducers and Activator of Transcription; type 5) [44]; PI3K/Akt (phosphatidyl inositol-3 kinase/Protein Kinase B) [45]; in some tissues including the heart, the MAPK (mitogen-activated protein kinase) pathway is involved [46]. MAPK and P13K/Akt inhibit caspase activation, thus directly attenuating apoptosis. We have shown that ARA 290 decreases cleaved caspase-3 in the myotubes subjected to ischaemia [18]. This may indicate that EPO and its derivatives may attenuate apoptosis through the MAPK or P13K/Akt pathway. However, further study is needed to understand completely the interactions of these signaling pathways that ultimately transduce tissue protection *in vivo*.

## 5. Discussion

It is now known that EPO receptors are present in human skeletal muscle and their expression is elevated in CLI. It has also been shown that a nonhaematopoietic derivative of EPO (ARA 290) decreases inflammation and apoptosis of ischaemic myotubes and may thus be used to selectively enhance the tissue-protective activity of EPO whilst avoiding the haematopoietic side effects in CLI.

The timing of administering EPO derivatives depends on their half-life. CEPO has a half-life of 4–6 hours while Asialo-EPO and ARA 290 have a half-life of about 2 minutes. Most *in vivo* studies have treated animals immediately following ischaemic injury [15, 23]. In the context of CLI, EPO derivatives may be used at the onset of CLI, either alone or as an adjunct to surgical or endovascular revascularization via systemic administration through intra-arterial, intravenous, or intramuscular routes. Due to the relatively short half-life of currently available compounds, repeated injections may be required. Clinical studies are needed to assess the efficacy of EPO derivatives in patients with CLI and to identify optimal delivery routes and dosing schedules.

## 6. Conclusion

EPO receptors are present in human skeletal muscle and their expression is elevated in CLI. Nonhaematopoietic derivatives of EPO decrease inflammation and apoptosis of ischaemic myotubes whilst avoiding haematopoietic side effects. They may thus reduce tissue damage and provide a unique therapeutic alternative for CLI.

## Figures and Tables

**Table 1:** Recent studies investigating the role of nonhaematopoietic derivatives of EPO.

Author	Nonhaematopoietic EPO derivative	Model	Inference
Erbayraktar et al. 2003 [10]	Asialo EPO	*In vivo* model of cerebral and spinal cord ischaemia plus sciatic nerve injury	Neuroprotection-recovery of paraplegia
Leis et al. 2004 [11]	Carbamylated EPO (CEPO)	*In vitro *and *in vivo *models of neural injury	Demonstration of tissue protective properties of CEPO in neural tissue
Fiordaliso et al. 2005 [12]	CEPO	*In vivo* model of rat myocardial infarction	Reduction in the size of myocardial infarct
Fantacci et al. 2006 [13]	CEPO	*In vivo *model of metabolic stress in mice induced by chronic hypoxia (CH)	CEPO reduced metabolic stress caused by CH and improved cellular survival independent of haematopoiesis.
Erbayraktar et al. 2006 [14]	CEPO	*In vivo *model of radiosurgically induced brain injury in rats	Decreased necrosis of the brain tissue
Erbayraktar et al. 2009 [15]	CEPO and ARA 290	*In vivo* models of decubitus ulcers and peritonitis	Improved healing and less intraperitoneal adhesion formation
Ahmet et al. 2010 [16]	ARA 290	*In vivo* model of myocardial ischemia	Reduction in the size of infarct and improvement of ejection fraction
Joshi et al. 2010 [17]	ARA 290	*In vitro*	Decreased apoptosis of myotubes but no effect on angiogenesis
Joshi et al. 2011 [18]	ARA 290	*In vitro*	Decreased ischaemia-induced inflammation and apoptosis of myotubes
